# Severe hyperglycemia and insulin resistance in patients with SARS-CoV-2 infection: a report of two cases

**DOI:** 10.1186/s40842-021-00121-y

**Published:** 2021-05-15

**Authors:** Alison H. Affinati, Amisha Wallia, Roma Y. Gianchandani

**Affiliations:** 1 Department of Internal Medicine, Division of Metabolism, Endocrinology and Diabetes, University of Michigan, Domino’s Farms (Lobby G, Suite 1500), 24 Frank Lloyd Wright Drive, MI 48106 Ann Arbor, USA; 2 Division of Endocrinology, Metabolism and Molecular Medicine, Northwestern University Feinberg School of Medicine, IL Chicago, USA

**Keywords:** Diabetes, COVID-19, Severe Insulin Resistance

## Abstract

**Background:**

Severe insulin resistance is an uncommon finding in patients with type 2 diabetes but is often associated with difficult to managing blood glucose. While severe insulin resistance is most frequently seen in the setting of medication side effects or rare genetic conditions, this report of two cases highlights the presence of severe insulin resistance in the setting of severe COVID-19 and explores how this may contribute to the poor prognosis of patients with diabetes who become infected with SARS-CoV-2.

**Case presentation:**

Here we present the cases of two African-American women with pre-existing type 2 diabetes who developed severe COVID-19 requiring mechanical ventilation and concurrent severe insulin resistance with total daily insulin dose requirements of greater than 5 unit/kg. Both patients received aggressive insulin infusion and subcutaneous insulin therapy to obtain adequate glucose management. As their COVID-19 clinical course improved, their severe insulin resistance improved as well.

**Conclusions:**

The association between critical illness and hyperglycemia is well documented in the literature, however severe insulin resistance is not commonly identified and may represent a unique clinical feature of the interaction between SARS-CoV-2 infection and type 2 diabetes.

## Introduction

SARS-CoV-2 infection is associated with severe hyperglycemia, which has been associated with poor patient outcomes [1–4]. While hyperglycemia is a feature of many severe infections, severe insulin resistance is less frequently documented. More commonly, severe insulin resistance is seen in the setting of medications or rare genetic disorders [5]. Here we present two cases of patients with severe Coronavirus Disease (COVID-19) who presented with insulin requirements greater than 5 units/kg. We use these cases as a starting point to explore the possible interactions between glucose homeostasis, SARS-CoV-2 infection and insulin treatment.

### Case report 1

A 41-year old African-American woman with a history of type 2 diabetes for at least 2 years, obesity (body mass index (BMI), 44 kg/m^2^), hypertension, hyperlipidemia and sleep apnea presented to the emergency department at an outside hospital with acute worsening of shortness of breath which had begun one week prior to presentation. On presentation she was hypoxic with associated confusion and was emergently intubated and mechanically ventilated. Her diabetes history was notable for a worsening of glucose control over the past two years, with her hemoglobin A1c increasing from 8.4 % to 2018 to 12.8 % in August of 2019 in the setting of medication non-adherence and ongoing depression. At the time of admission, her hemoglobin A1c was 11.6 %. Chart review indicated that she was prescribed Lantus 60 units daily, metformin 1000 mg twice daily, glimepiride 4 mg twice daily with meals and 1.5 mg dulaglutide weekly as an outpatient. Physical exam was notable for acanthosis nigricans.

On admission, laboratory testing was notable for a sodium of 130 mEq/L, potassium 5.0 mEq/L, chloride 93 mmol/L, bicarbonate 16 mmol/L (anion gap 21), blood glucose 760 mg/dL, creatinine 1.65 mg/dL (eGFR 44), lactic acid of 8.33 mmol/L and serum β-hydroxybutyrate of 2.69 mmol/L. Arterial blood gas showed a pH of 7.36, pCO_2_ of 31 mmHg and pO_2_ of 55 mmHg, consistent with a mixed anion gap metabolic acidosis and respiratory alkalosis. SARS-CoV-2 PCR testing was positive. See Table 1 for additional laboratory values, including inflammatory markers, which were initially elevated on admission.

**Table 1:** Laboratory values measured on admission

Laboratory Test	Patient 1	Patient 2
WBC (cells x10^3^/uL)	19.9	18.34
Neutrophils (cells x10^3^/uL)	17.3	14.12
Lymphocytes (cells x10^3^/uL)	0.8	2.57
Monocytes (cells x10^3^/uL)	1.9	1.28
Hemoglobin (g/dL)	13.7	11.6
Serum Chemistries		
Sodium (mEq/L)	130	141
Potassium (mEq/L)	5	4.6
Chloride (mEq/L)	93	100
Bicarbonate (mEq/L)	16	27
BUN (mg/dL)	19	74
Creatinine (mg/dL)	1.65	2.01
Glucose (mg/dL)	760	272
Anion Gap	21	14
Calcium (mg/dL)	8.7	9.2
Albumin (g/dL)	3.9	3.8
Alkaline Phosphatase (IU/L)	105	98
AST (units/L)	81	44
ALT (units/L)	44	69
Total Bilirubin (mg/dL)	1.1	<0.2
Arterial Blood Gas		
Arterial pH	7.36	7.4
Arterial CO_2_ (mmHg)	31	56
Arterial O_2_ (mmHg)	55	68
Inflammatory Markers		
ESR (mm/hr)	85	120
CRP (mg/L)	128.5	121.7
CK (units/L)	-	82
Fibrinogen (mg/dL)	669	295 (transfer)
LDH (units/L)		
Admission	810	885
Transfer	-	871
Procalcitonin (ng/mL)		
Admission	-	0.72
Transfer	0.17	0.21
D-Dimer (ng/mL)		
Admission	1547	969
Transfer	12.19	> 35
Ferritin (ng/mL)		
Admission	98	744
Transfer	-	595.3
Triglycerides (mg/dL)		
Admission	352	166
Transfer	347	295
Hemoglobin A1c	11.60%	7.30%

**Fig. 1:**
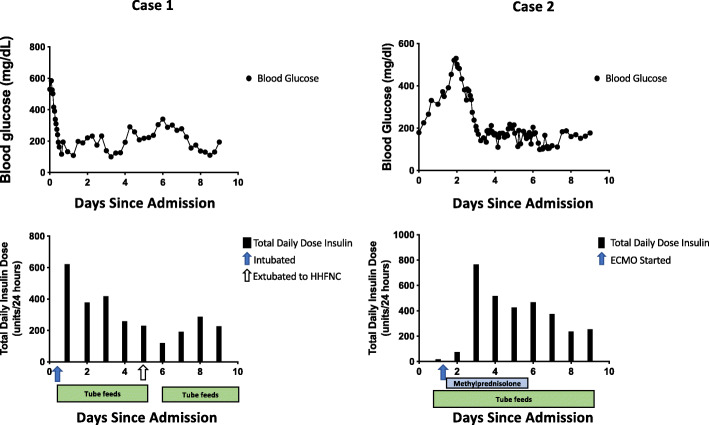
Glucose levels and total daily insulin dose during the first 10 days following admission. Glucose levels (black circles, upper panel) and total daily dose of insulin (black bars, lower panel) by days following admission. Respiratory interventions indicated by arrows. Blue bars represent duration of 1 mg/kg methylprednisolone treatment for ARDS. Green bars represent duration of tube feeds

Due to the elevated serum ketones and anion-gap metabolic acidosis, she was treated for diabetic ketoacidosis (DKA) with a continuous insulin infusion at 7 units/hr (0.05 units/kg/hr) along with a normal saline infusion. Her blood glucose levels remained elevated in the 400–500 mg/dL range while her anion gap decreased to 15 but did not resolve. Over the next 36 h her insulin infusion rate peaked at 34.5 units/hr (0.26 units/kg/hr) and she was started on 50 units of insulin glargine and 50 units of regular insulin every 6 h in addition to the insulin infusion to facilitate weaning off of the insulin drip. During the first 36 h following transfer to our hospital, she had an average insulin requirement of 5 units/kg/day. Her insulin infusion was weaned off within 48 h and her subcutaneous insulin requirements continued to be elevated at 3.24 units/kg/day. Following extubation on day 6, her insulin requirements dropped to 211 units per day (1.64 units/kg) and she continued to require over 200 units of subcutaneous insulin daily for the next 13 days (Fig. 1). Her insulin requirements improved as she was weaned off of heated high flow nasal cannula and transitioned from tube feeds to a diabetic diet. Prior to discharge to a subacute rehabilitation center, she was transitioned to her home regimen of 60 units Lantus, metformin, glimepiride and dulaglutide. While in the hospital she had well-controlled blood sugars on this regimen. Two months after discharge, while still at the subacute rehabilitation center, her hemoglobin A1c was 6.0 %. Her kidney function had returned to her baseline (eGFR > 90).

### Case 2

A 47-year old African-American woman with a history of pre-diabetes on metformin, obesity (BMI 39 kg/m^2^), hypertension and hyperlipidemia presented with cough, dyspnea, hypoxia and fever and was emergently intubated. She was found to be SARS-CoV-2 positive. She was initially treated with hydroxychloroquine, azithromycin, 40 mg methylprednisolone twice daily and high dose vitamin C with blood glucoses recorded in the 300 s. Her hemoglobin A1c was 7.3 % on admission.

Regarding her diabetes history, she was diagnosed with pre-diabetes in 2016 with a hemoglobin A1c of 5.9 % and started on metformin therapy. Her hemoglobin A1c ranged between 5.5 and 6.2 % until her admission for COVID-19.

Following admission, her course was complicated by significant difficulty with oxygenation, pneumomediastinum due to barotrauma and acute kidney injury. She was ultimately transferred to our institution for extra-corporeal membrane oxygenation (ECMO) from an outside hospital. On arrival at our hospital, she received 1 dose of sarilumab (human monoclonal antibody against interleukin-6 (IL-6)) and was started on IV methylprednisolone. She rapidly developed worsening hyperglycemia with glucose levels above 400 mg/dL in the setting of methylprednisolone treatment and was started on an insulin infusion. Subcutaneous insulin was initiated simultaneously to assist in weaning off the insulin infusion as rapidly as possible. This protocol has recently been published in *Gianchandani et al.* [6]. Over the next 24 h she required insulin drip rates up to 45 units/hr in addition to 20 units insulin glargine twice daily and 16 units regular insulin every 6 h (see Fig. 1). The subcutaneous insulin was titrated up over 4 days until she was receiving 30 units of insulin glargine twice a day and 56 units of regular insulin every 6 h with drip rates between 2 and 10 units/hr. Her 24-hour insulin requirements peaked at 6.45 units/kg/day. After discontinuation of methylprednisolone her insulin requirements decreased significantly, her insulin infusion was titrated off and subcutaneous insulin was decreased to Lantus 25 units twice daily and regular insulin 48 units every 6 h. Despite this improvement, she did continue to require over 200 units of insulin daily via subcutaneous injection even after discontinuation of the methylprednisolone. Six days after methylprednisolone discontinuation, her insulin requirements dropped precipitously, which coincided with ECMO decannulation. Insulin was discontinued completely 12 days later. She continued to require mechanical ventilation for over 2 months and was discharged to a long-term care facility for further ventilator weaning. Six months after discharge from the hospital, her creatinine had not yet returned to baseline, but had improved to 1.12 (eGFR 58). Her hemoglobin A1c at that time was 5.9 % without any diabetes medications. She had successfully been liberated from the ventilator and returned home.

## Discussion

Over the last year, a picture has emerged that illustrates the increased risk of severe infection and death in patients with diabetes who develop COVID-19. Almost 30 % of patients with diabetes who present to the hospital with COVID-19 require intubation and over 10 % will die within a week [1]. Patients with diabetes are more likely to require hospitalization and ICU admission and have a higher risk of death than those without diabetes [2–4]. Here we present the cases of two patients with a history of diabetes that developed COVID-19, difficult to control hyperglycemia and severe insulin resistance, prompting us to consider the mechanisms underlying the poor prognosis for patients with diabetes and how glucose control and insulin treatment might interact with this infection.

Prior to the COVID-19 pandemic, there was already a wealth of data demonstrating that hyperglycemia and insulin resistance during critical illness is associated with worse outcomes [7–10]. One of the remarkable features of the patients presented here is the degree of insulin resistance, with both patients requiring more than 5 units/kg/day shortly after admission. This meets the criteria for extreme insulin resistance, defined as more than 3 units/kg/day. Extreme insulin resistance is most often seen in the setting of rare genetic disorders such as lipodystrophy and severe insulin resistance syndrome, use of medications such as glucocorticoids or endocrinopathies such as Cushings [5].

As dexamethasone is one of the few treatments that decreases mortality in severe COVID-19 infection [11, 12], this is likely a significant contributor to the severe hyperglycemia in many patients with COVID-19. However, it is not the only cause, as the first patient described in this report developed severe hyperglycemia and insulin resistance despite never receiving steroids. Similarly, the second patient continued to have severe insulin resistance for 6 days following discontinuation of steroids.

Acute infections also lead to increased insulin resistance through enhanced secretion of the counter-regulatory hormones cortisol, glucagon and growth hormone [13]. In addition to the hormonal response to stress, cytokine expression may independently contribute to increasing insulin resistance during infection [14]. Thus, the robust inflammatory response in patients with SARS-CoV-2 could be a significant contributor to their severe insulin resistance [6], as both of our patients had elevated inflammatory markers that coincided with increasing insulin demands (Table 1).

The interaction between insulin resistance, severe hyperglycemia and inflammation is likely to be particularly important in the setting of SARS-CoV-2 infection, as the robust inflammatory response has been linked to poor outcomes [15]. In patients with diabetes who present with COVID-19, the inflammatory marker C-reactive protein (CRP) has been identified as one of the strongest risk factors for mortality [2], while patients with prolonged hyperglycemia have higher IL-6 and D-dimer levels and a higher risk of progressing to severe disease [16]. While inflammation leads to hyperglycemia and insulin resistance, hyperglycemia and insulin resistance also exacerbate inflammation, impair immune cell function and promote epithelial cell dysfunction, even in otherwise healthy volunteers [17–21].

Treatment of hyperglycemia in patients with COVID-19 has been particularly difficult as many centers have attempted to limit insulin infusions to preserve PPE and minimize health care provider exposure, as treatment with insulin infusion require hourly finger stick blood glucose checks. Given the extraordinary doses of insulin required in the patients presented here, it is valuable to consider the potential effects of insulin beyond its hypoglycemic effects, and in particular, its role in inflammation.

Insulin’s role in inflammation is likely to be context dependent. When evaluated in the setting of obesity-related insulin resistance, high doses of insulin may be pro-inflammatory. In both obese mice and humans, hyperinsulinemia results in elevated adipose tissue cytokine levels [22–25], while this does not occur in healthy, non-obese and non-insulin resistant patients [26]. In contrast, many studies have demonstrated that insulin treatment in the setting of sepsis is anti-inflammatory. In multiple animal models of infection, insulin treatment decreases systemic cytokines independent of glucose levels [27–29]. This finding holds true in human studies as well. In healthy patients with both type 1 and type 2 diabetes, insulin infusion (with dextrose to maintain euglycemia) leads to decreased reactive oxygen species, systemic cytokine gene expression and serum CRP levels [30–32].

## Conclusions

Based on our review of the literature and exemplified by the cases presented here, it is likely that hyperglycemia in patients with COVID-19 infection worsens the dramatic inflammation and cytokine storm that accompanies severe infection. Insulin treatment reduces blood glucose levels and may have an anti-inflammatory action in the setting of COVID-19 sepsis.

## Data Availability

Data sharing is not applicable to this case report as no datasets were generated or analyzed during the current study.

## Abbreviations

COVID-19: Coronavirus Disease 2019
DKA: Diabetic ketoacidosis
ECMO: Extra-corporeal membrane oxygenation
CRP: C-reactive protein
IL-6: Interleukin-6
